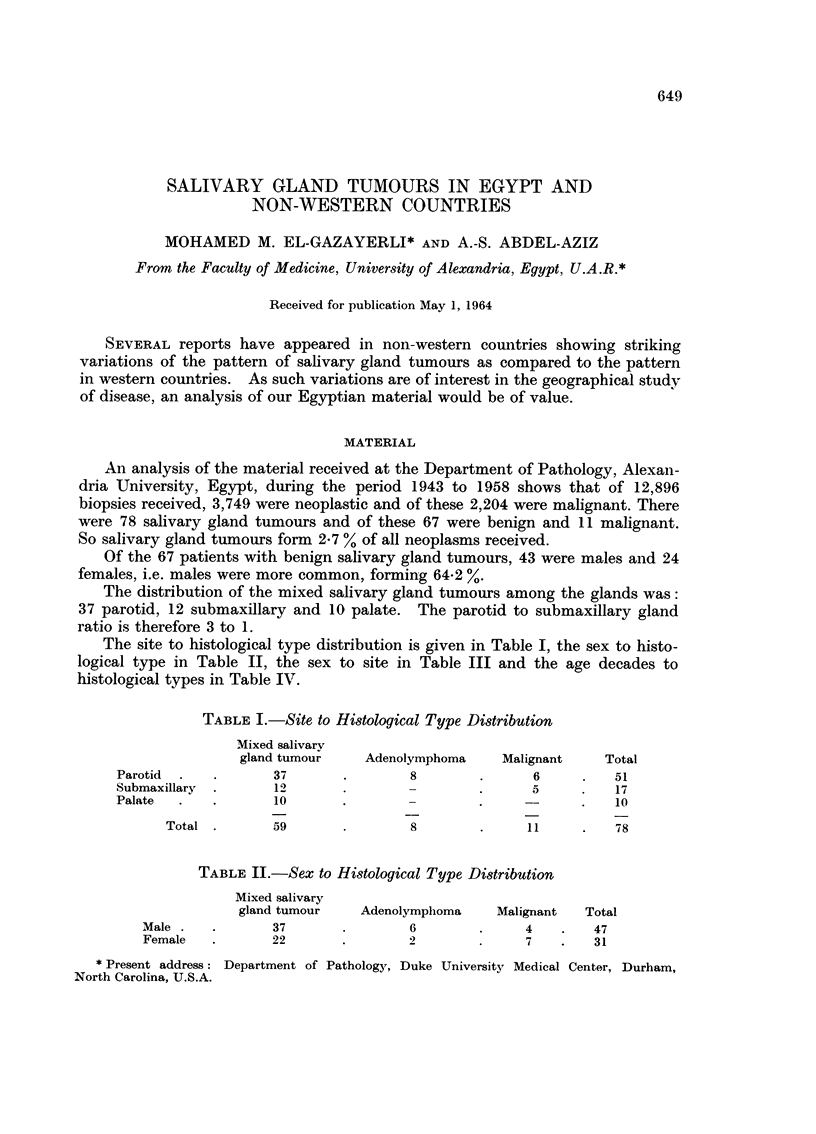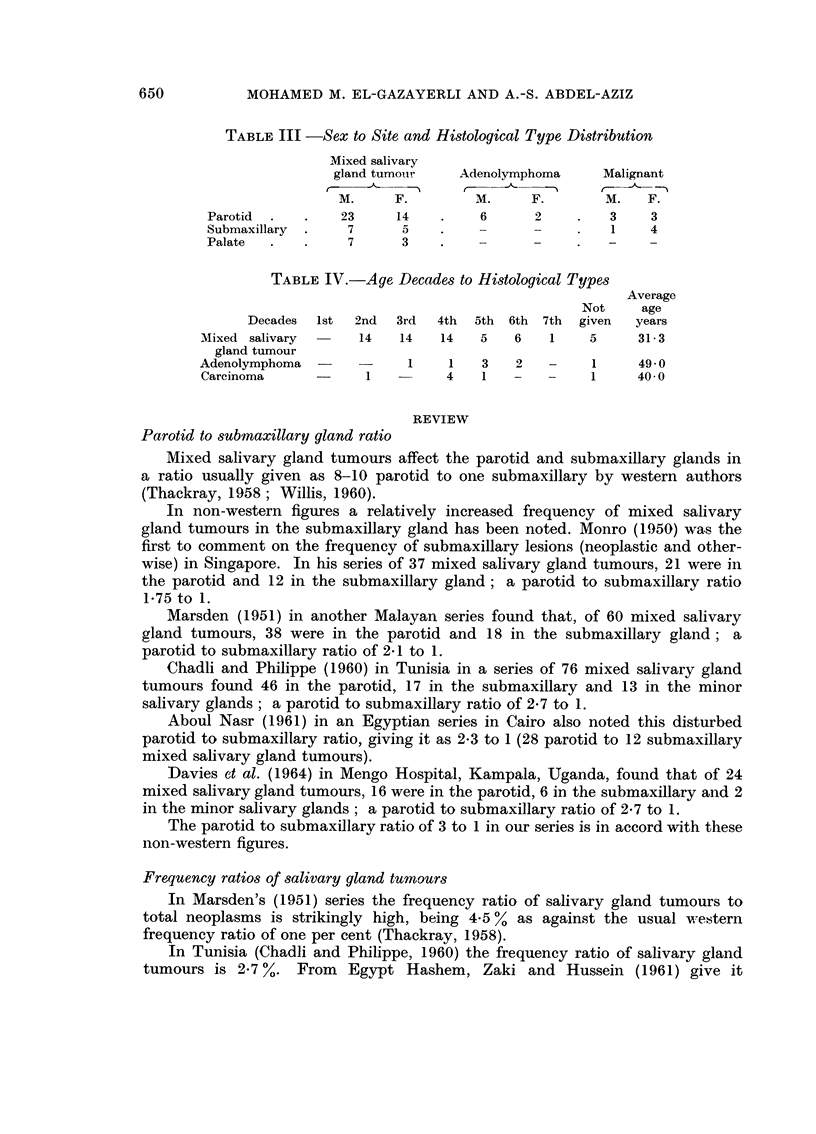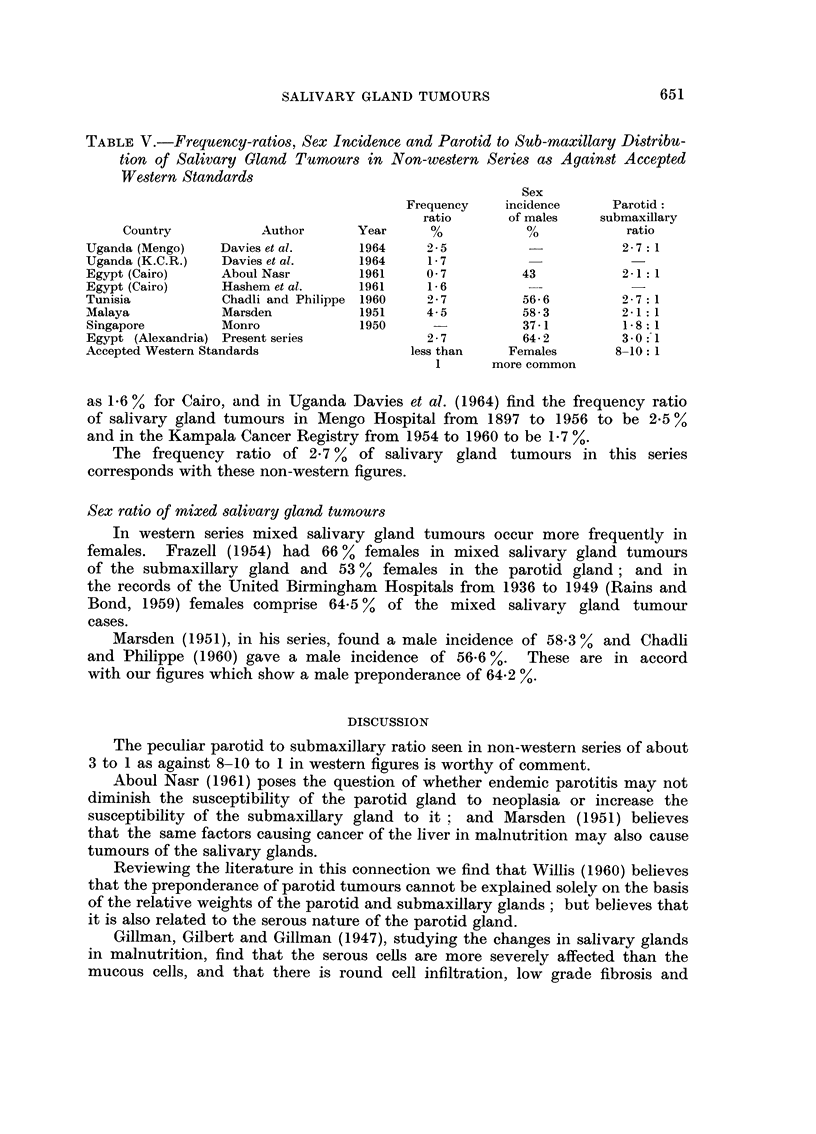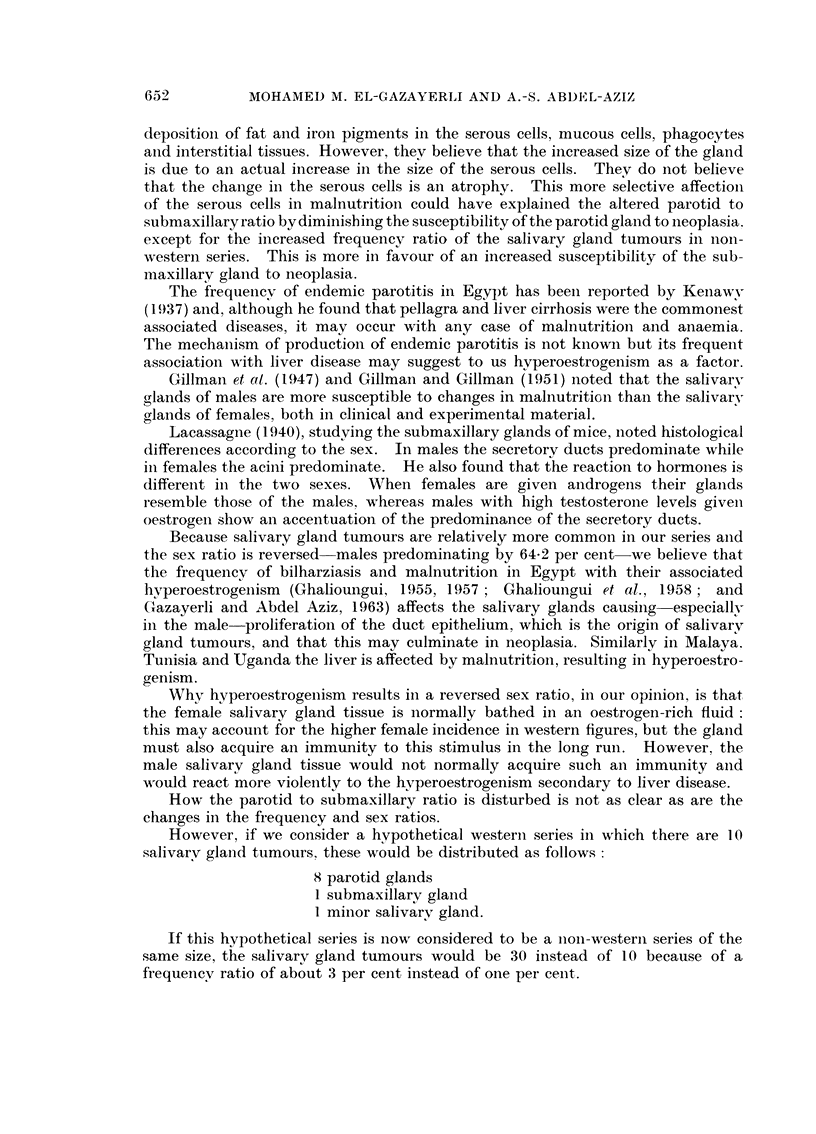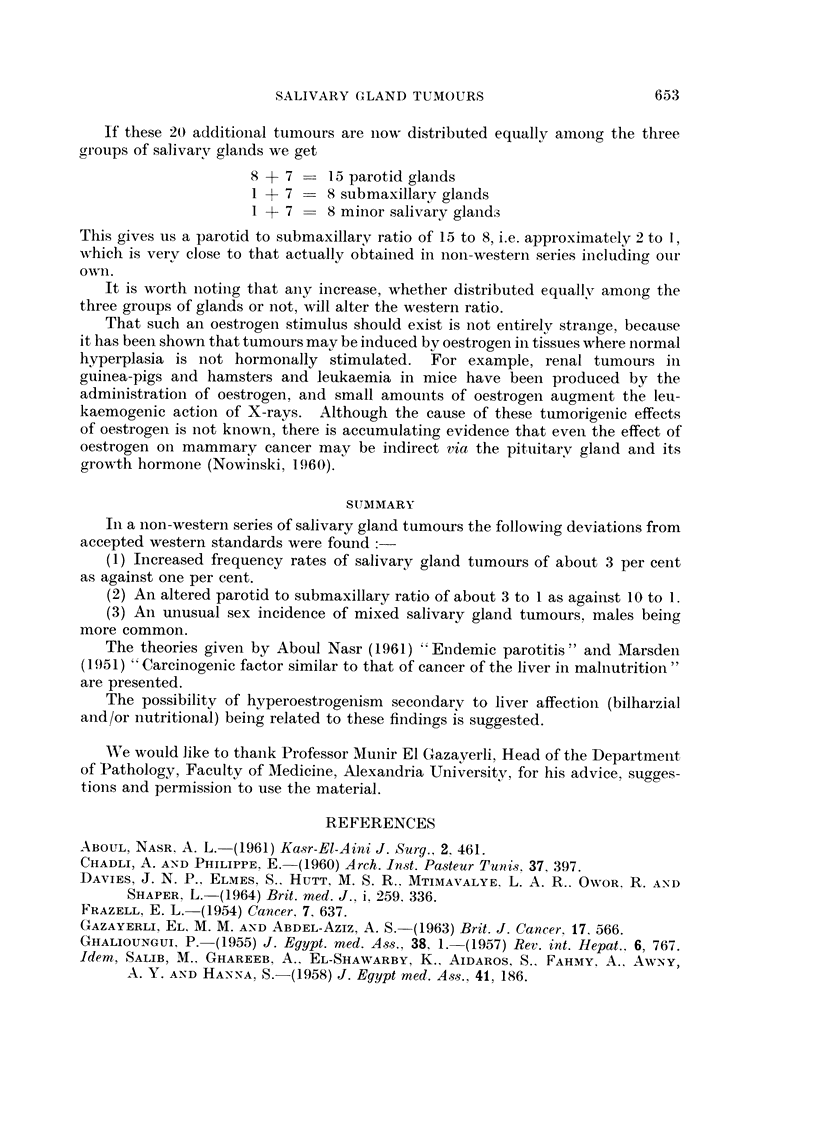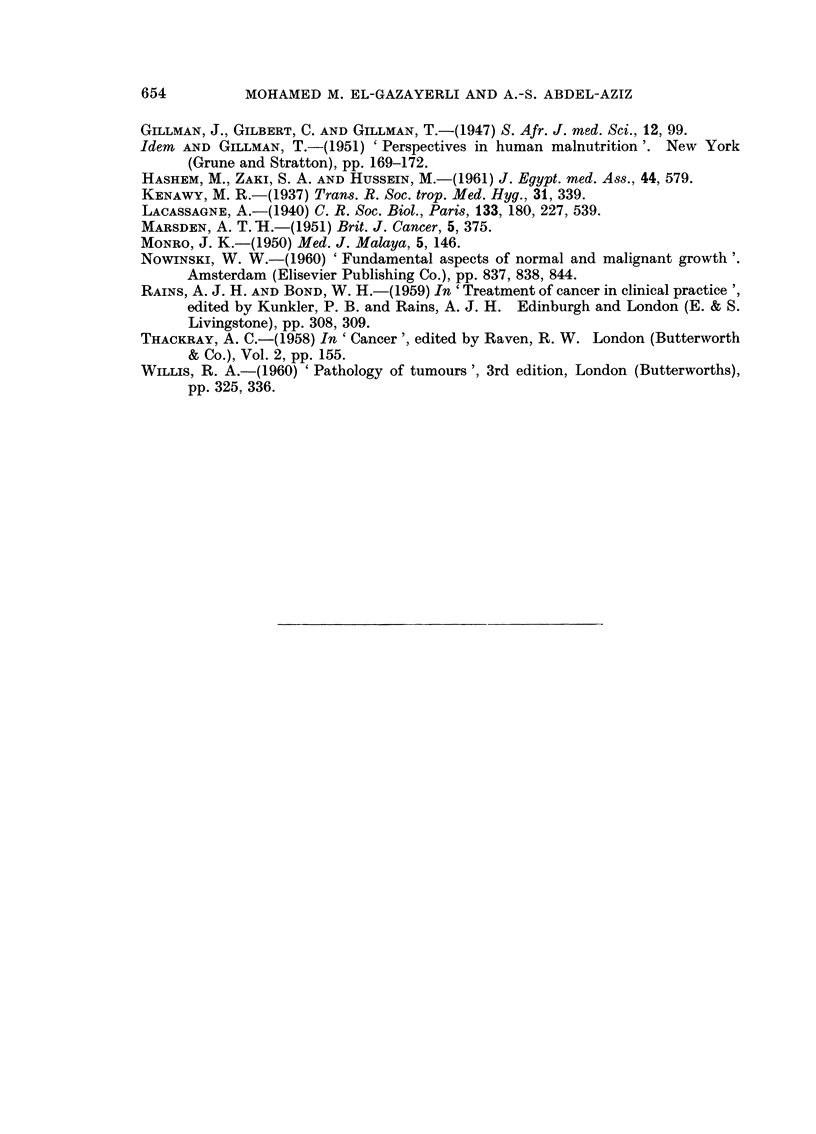# Salivary Gland Tumours in Egypt and Non-Western Countries

**DOI:** 10.1038/bjc.1964.74

**Published:** 1964-12

**Authors:** Mohamed M. El-Gazayerli, A.-S. Abdel-Aziz


### SALIVARY GLAND TUMOURS IN EGYPT AND NON-WESTERN COUNTRIES

MOHAMED M. EL-GAZAYERLI* AND A.-S. ABDEL-AZIZ

*From the Faculty of Medicine, University of Alexandria, Egypt, U.A.R.**

Received for publication May 1, 1964

SEVERAL reports have appeared in non-western countries showing striking variations of the pattern of salivary gland tumours as compared to the pattern in western countries. As such variations are of interest in the geographical study of disease, an analysis of our Egyptian material would be of value.

### MATERIAL

An analysis of the material received at the Department of Pathology, Alexandria University, Egypt, during the period 1943 to 1958 shows that of 12,896 biopsies received, 3,749 were neoplastic and of these 2,204 were malignant. There were 78 salivary gland tumours and of these 67 were benign and 11 malignant. So salivary gland tumours form 2·7 % of all neoplasms received.

Of the 67 patients with benign salivary gland tumours, 43 were males and 24 females, i.e. males were more common, forming 64·2 %.

The distribution of the mixed salivary gland tumours among the glands was: 37 parotid, 12 submaxillary and 10 palate. The parotid to submaxillary gland ratio is therefore 3 to 1.

The site to histological type distribution is given in Table I, the sex to histological type in Table II, the sex to site in Table III and the age decades to histological types in Table IV.

TABLE I.—*Site to Histological Type Distribution*

| | Mixed salivary gland tumour | Adenolymphoma | Malignant | Total |
|---|---|---|---|---|
| Parotid | 37 | 8 | 6 | 51 |
| Submaxillary | 12 | – | 5 | 17 |
| Palate | 10 | – | — | 10 |
| Total | 59 | 8 | 11 | 78 |

TABLE II.—*Sex to Histological Type Distribution*

| | Mixed salivary gland tumour | Adenolymphoma | Malignant | Total |
|---|---|---|---|---|
| Male | 37 | 6 | 4 | 47 |
| Female | 22 | 2 | 7 | 31 |

* Present address: Department of Pathology, Duke University Medical Center, Durham, North Carolina, U.S.A.

TABLE III —*Sex to Site and Histological Type Distribution*

| | Mixed salivary gland tumour | | Adenolymphoma | | Malignant | |
|---|---|---|---|---|---|---|
| | M. | F. | M. | F. | M. | F. |
| Parotid | 23 | 14 | 6 | 2 | 3 | 3 |
| Submaxillary | 7 | 5 | – | – | 1 | 4 |
| Palate | 7 | 3 | – | – | – | – |

TABLE IV.—*Age Decades to Histological Types*

| Decades | 1st | 2nd | 3rd | 4th | 5th | 6th | 7th | Not given | Average age years |
|---|---|---|---|---|---|---|---|---|---|
| Mixed salivary gland tumour | — | 14 | 14 | 14 | 5 | 6 | 1 | 5 | 31·3 |
| Adenolymphoma | — | — | 1 | 1 | 3 | 2 | – | 1 | 49·0 |
| Carcinoma | — | 1 | — | 4 | 1 | – | – | 1 | 40·0 |

### REVIEW

*Parotid to submaxillary gland ratio*

Mixed salivary gland tumours affect the parotid and submaxillary glands in a ratio usually given as 8–10 parotid to one submaxillary by western authors (Thackray, 1958 ; Willis, 1960).

In non-western figures a relatively increased frequency of mixed salivary gland tumours in the submaxillary gland has been noted. Monro (1950) was the first to comment on the frequency of submaxillary lesions (neoplastic and otherwise) in Singapore. In his series of 37 mixed salivary gland tumours, 21 were in the parotid and 12 in the submaxillary gland ; a parotid to submaxillary ratio 1·75 to 1.

Marsden (1951) in another Malayan series found that, of 60 mixed salivary gland tumours, 38 were in the parotid and 18 in the submaxillary gland ; a parotid to submaxillary ratio of 2·1 to 1.

Chadli and Philippe (1960) in Tunisia in a series of 76 mixed salivary gland tumours found 46 in the parotid, 17 in the submaxillary and 13 in the minor salivary glands ; a parotid to submaxillary ratio of 2·7 to 1.

Aboul Nasr (1961) in an Egyptian series in Cairo also noted this disturbed parotid to submaxillary ratio, giving it as 2·3 to 1 (28 parotid to 12 submaxillary mixed salivary gland tumours).

Davies *et al.* (1964) in Mengo Hospital, Kampala, Uganda, found that of 24 mixed salivary gland tumours, 16 were in the parotid, 6 in the submaxillary and 2 in the minor salivary glands ; a parotid to submaxillary ratio of 2·7 to 1.

The parotid to submaxillary ratio of 3 to 1 in our series is in accord with these non-western figures.

*Frequency ratios of salivary gland tumours*

In Marsden's (1951) series the frequency ratio of salivary gland tumours to total neoplasms is strikingly high, being 4·5% as against the usual western frequency ratio of one per cent (Thackray, 1958).

In Tunisia (Chadli and Philippe, 1960) the frequency ratio of salivary gland tumours is 2·7%. From Egypt Hashem, Zaki and Hussein (1961) give it

TABLE V.—*Frequency-ratios, Sex Incidence and Parotid to Sub-maxillary Distribution of Salivary Gland Tumours in Non-western Series as Against Accepted Western Standards*

| Country | Author | Year | Frequency ratio % | Sex incidence of males % | Parotid : submaxillary ratio |
|---|---|---|---|---|---|
| Uganda (Mengo) | Davies *et al.* | 1964 | 2·5 | — | 2·7 : 1 |
| Uganda (K.C.R.) | Davies *et al.* | 1964 | 1·7 | — | — |
| Egypt (Cairo) | Aboul Nasr | 1961 | 0·7 | 43 | 2·1 : 1 |
| Egypt (Cairo) | Hashem *et al.* | 1961 | 1·6 | — | — |
| Tunisia | Chadli and Philippe | 1960 | 2·7 | 56·6 | 2·7 : 1 |
| Malaya | Marsden | 1951 | 4·5 | 58·3 | 2·1 : 1 |
| Singapore | Monro | 1950 | — | 37·1 | 1·8 : 1 |
| Egypt (Alexandria) | Present series | | 2·7 | 64·2 | 3·0 : 1 |
| Accepted Western Standards | | | less than 1 | Females more common | 8–10 : 1 |

as 1·6 % for Cairo, and in Uganda Davies *et al.* (1964) find the frequency ratio of salivary gland tumours in Mengo Hospital from 1897 to 1956 to be 2·5 % and in the Kampala Cancer Registry from 1954 to 1960 to be 1·7 %.

The frequency ratio of 2·7 % of salivary gland tumours in this series corresponds with these non-western figures.

*Sex ratio of mixed salivary gland tumours*

In western series mixed salivary gland tumours occur more frequently in females. Frazell (1954) had 66 % females in mixed salivary gland tumours of the submaxillary gland and 53 % females in the parotid gland ; and in the records of the United Birmingham Hospitals from 1936 to 1949 (Rains and Bond, 1959) females comprise 64·5 % of the mixed salivary gland tumour cases.

Marsden (1951), in his series, found a male incidence of 58·3 % and Chadli and Philippe (1960) gave a male incidence of 56·6 %. These are in accord with our figures which show a male preponderance of 64·2 %.

### DISCUSSION

The peculiar parotid to submaxillary ratio seen in non-western series of about 3 to 1 as against 8–10 to 1 in western figures is worthy of comment.

Aboul Nasr (1961) poses the question of whether endemic parotitis may not diminish the susceptibility of the parotid gland to neoplasia or increase the susceptibility of the submaxillary gland to it ; and Marsden (1951) believes that the same factors causing cancer of the liver in malnutrition may also cause tumours of the salivary glands.

Reviewing the literature in this connection we find that Willis (1960) believes that the preponderance of parotid tumours cannot be explained solely on the basis of the relative weights of the parotid and submaxillary glands ; but believes that it is also related to the serous nature of the parotid gland.

Gillman, Gilbert and Gillman (1947), studying the changes in salivary glands in malnutrition, find that the serous cells are more severely affected than the mucous cells, and that there is round cell infiltration, low grade fibrosis and deposition of fat and iron pigments in the serous cells, mucous cells, phagocytes and interstitial tissues. However, they believe that the increased size of the gland is due to an actual increase in the size of the serous cells. They do not believe that the change in the serous cells is an atrophy. This more selective affection of the serous cells in malnutrition could have explained the altered parotid to submaxillary ratio by diminishing the susceptibility of the parotid gland to neoplasia, except for the increased frequency ratio of the salivary gland tumours in non-western series. This is more in favour of an increased susceptibility of the submaxillary gland to neoplasia.

The frequency of endemic parotitis in Egypt has been reported by Kenawy (1937) and, although he found that pellagra and liver cirrhosis were the commonest associated diseases, it may occur with any case of malnutrition and anaemia. The mechanism of production of endemic parotitis is not known but its frequent association with liver disease may suggest to us hyperoestrogenism as a factor.

Gillman *et al.* (1947) and Gillman and Gillman (1951) noted that the salivary glands of males are more susceptible to changes in malnutrition than the salivary glands of females, both in clinical and experimental material.

Lacassagne (1940), studying the submaxillary glands of mice, noted histological differences according to the sex. In males the secretory ducts predominate while in females the acini predominate. He also found that the reaction to hormones is different in the two sexes. When females are given androgens their glands resemble those of the males, whereas males with high testosterone levels given oestrogen show an accentuation of the predominance of the secretory ducts.

Because salivary gland tumours are relatively more common in our series and the sex ratio is reversed—males predominating by 64·2 per cent—we believe that the frequency of bilharziasis and malnutrition in Egypt with their associated hyperoestrogenism (Ghalioungui, 1955, 1957 ; Ghalioungui *et al.*, 1958 ; and Gazayerli and Abdel Aziz, 1963) affects the salivary glands causing—especially in the male—proliferation of the duct epithelium, which is the origin of salivary gland tumours, and that this may culminate in neoplasia. Similarly in Malaya, Tunisia and Uganda the liver is affected by malnutrition, resulting in hyperoestrogenism.

Why hyperoestrogenism results in a reversed sex ratio, in our opinion, is that the female salivary gland tissue is normally bathed in an oestrogen-rich fluid ; this may account for the higher female incidence in western figures, but the gland must also acquire an immunity to this stimulus in the long run. However, the male salivary gland tissue would not normally acquire such an immunity and would react more violently to the hyperoestrogenism secondary to liver disease.

How the parotid to submaxillary ratio is disturbed is not as clear as are the changes in the frequency and sex ratios.

However, if we consider a hypothetical western series in which there are 10 salivary gland tumours, these would be distributed as follows :

8 parotid glands

1 submaxillary gland

1 minor salivary gland.

If this hypothetical series is now considered to be a non-western series of the same size, the salivary gland tumours would be 30 instead of 10 because of a frequency ratio of about 3 per cent instead of one per cent.

If these 20 additional tumours are now distributed equally among the three groups of salivary glands we get

$8 + 7 = 15$ parotid glands

$1 + 7 = 8$ submaxillary glands

$1 + 7 = 8$ minor salivary glands

This gives us a parotid to submaxillary ratio of 15 to 8, i.e. approximately 2 to 1, which is very close to that actually obtained in non-western series including our own.

It is worth noting that any increase, whether distributed equally among the three groups of glands or not, will alter the western ratio.

That such an oestrogen stimulus should exist is not entirely strange, because it has been shown that tumours may be induced by oestrogen in tissues where normal hyperplasia is not hormonally stimulated. For example, renal tumours in guinea-pigs and hamsters and leukaemia in mice have been produced by the administration of oestrogen, and small amounts of oestrogen augment the leukaemogenic action of X-rays. Although the cause of these tumorigenic effects of oestrogen is not known, there is accumulating evidence that even the effect of oestrogen on mammary cancer may be indirect *via* the pituitary gland and its growth hormone (Nowinski, 1960).

### SUMMARY

In a non-western series of salivary gland tumours the following deviations from accepted western standards were found :—

(1) Increased frequency rates of salivary gland tumours of about 3 per cent as against one per cent.

(2) An altered parotid to submaxillary ratio of about 3 to 1 as against 10 to 1.

(3) An unusual sex incidence of mixed salivary gland tumours, males being more common.

The theories given by Aboul Nasr (1961) "Endemic parotitis" and Marsden (1951) "Carcinogenic factor similar to that of cancer of the liver in malnutrition" are presented.

The possibility of hyperoestrogenism secondary to liver affection (bilharzial and/or nutritional) being related to these findings is suggested.

We would like to thank Professor Munir El Gazayerli, Head of the Department of Pathology, Faculty of Medicine, Alexandria University, for his advice, suggestions and permission to use the material.

### REFERENCES

Aboul, Nasr, A. L.—(1961) *Kasr-El-Aini J. Surg.*, **2**, 461.
Chadli, A. and Philippe, E.—(1960) *Arch. Inst. Pasteur Tunis*, **37**, 397.
Davies, J. N. P., Elmes, S., Hutt, M. S. R., Mtimavalye, L. A. R., Owor, R. and Shaper, L.—(1964) *Brit. med. J.*, i, 259, 336.
Frazell, E. L.—(1954) *Cancer*, **7**, 637.
Gazayerli, El, M. M. and Abdel-Aziz, A. S.—(1963) *Brit. J. Cancer*, **17**, 566.
Ghalioungui, P.—(1955) *J. Egypt. med. Ass.*, **38**, 1.—(1957) *Rev. int. Hepat.*, **6**, 767.
*Idem*, Salib, M., Ghareeb, A., El-Shawarby, K., Aidaros, S., Fahmy, A., Awny, A. Y. and Hanna, S.—(1958) *J. Egypt med. Ass.*, **41**, 186.

GILLMAN, J., GILBERT, C. AND GILLMAN, T.—(1947) *S. Afr. J. med. Sci.*, **12**, 99.

*Idem* AND GILLMAN, T.—(1951) 'Perspectives in human malnutrition'. New York (Grune and Stratton), pp. 169–172.

HASHEM, M., ZAKI, S. A. AND HUSSEIN, M.—(1961) *J. Egypt. med. Ass.*, **44**, 579.

KENAWY, M. R.—(1937) *Trans. R. Soc. trop. Med. Hyg.*, **31**, 339.

LACASSAGNE, A.—(1940) *C. R. Soc. Biol., Paris*, **133**, 180, 227, 539.

MARSDEN, A. T. H.—(1951) *Brit. J. Cancer*, **5**, 375.

MONRO, J. K.—(1950) *Med. J. Malaya*, **5**, 146.

NOWINSKI, W. W.—(1960) 'Fundamental aspects of normal and malignant growth'. Amsterdam (Elisevier Publishing Co.), pp. 837, 838, 844.

RAINS, A. J. H. AND BOND, W. H.—(1959) *In* 'Treatment of cancer in clinical practice', edited by Kunkler, P. B. and Rains, A. J. H. Edinburgh and London (E. & S. Livingstone), pp. 308, 309.

THACKRAY, A. C.—(1958) *In* 'Cancer', edited by Raven, R. W. London (Butterworth & Co.), Vol. 2, pp. 155.

WILLIS, R. A.—(1960) 'Pathology of tumours', 3rd edition, London (Butterworths), pp. 325, 336.